# 307 Reducing Moral Distress in Burn/trauma Intensive Care Unit Nurses

**DOI:** 10.1093/jbcr/irad045.282

**Published:** 2023-08-29

**Authors:** Mikayla B Miller

**Affiliations:** Medstar Washington Hospital Center, WOODBRIDGE, Virginia

## Abstract

**Introduction:**

There is minimal research regarding moral distress in Burn/Trauma intensive care unit (ICU) nurses compared to other nursing specialties. However, there is evidence that indicates there are high levels of moral distress in this nursing population which increases the risk for psychological or physical distress, burnout, and high nursing turnover. Identifying and utilizing an effective intervention, such as nursing huddles with clinical ethicists, can help reduce the impact of moral distress in this nursing population.

**Methods:**

An evidenced-based practice project using the IOWA Model was conducted over a six week period on a ten bed Burn/Trauma ICU. Convenience sampling was utilized to capture a total of 24 registered nurses (RN) in the initial survey period. Each of the nurses (n=24) completed the Moral Distress Thermometer (MDT) and the Measure of Moral Distress for Healthcare Providers (MMD-HP) prior to signing up for a nursing huddle with the clinical ethicists. There were five nursing huddles conducted with clinical ethicists on Microsoft Teams over the span of four weeks. Each nurse that participated was required to complete the pre-intervention surveys prior to participating in the nursing huddles and also complete the post MDT evaluation. The MDT pre and post-intervention scores were compared to evaluate the impact of nursing huddles with clinical ethicists on moral distress among RNs working in a Burn/Trauma ICU.

**Results:**

Out of the 24 RNs, 62.5% (n=15) completed the pre-intervention surveys, nursing huddles, and post-intervention surveys. A comparison of the pre-intervention and post-intervention MDT scores showed approximately a 44% decrease in acute moral distress in the Burn/Trauma ICU nurses. In addition, the MMD-HP identified 10 consistent triggers of moral distress in all 24 RNs during the initial survey period. Some reoccurring themes included futile care, false hope, and unnecessary care with benefit to the patient.

**Conclusions:**

Nursing huddles with clinical ethicists considerably reduced the impact of the moral distress identified in Burn/Trauma ICU nurses. These findings support the need for continuous evaluation of moral distress and implementation of interventions to reduce moral distress in this nursing specialty.

**Applicability of Research to Practice:**

Identifying and mitigating moral distress in the Burn/Trauma ICU nurse population is absolutely necessary. The unique ethical dilemmas that arise on a daily basis requires continuous monitoring and implementation of varying interventions to support the nurses and reduce the level of moral distress they may be experiencing. Moral distress does not dissipate on its own. Moral distress needs to be addressed and recognized as a serious and prevalent issue in this nursing population.

